# Fabry Disease: More than a Phenocopy of Hypertrophic Cardiomyopathy

**DOI:** 10.3390/jcm12227061

**Published:** 2023-11-13

**Authors:** Kamil Stankowski, Stefano Figliozzi, Vincenzo Battaglia, Federica Catapano, Marco Francone, Lorenzo Monti

**Affiliations:** 1 Department of Biomedical Sciences, Humanitas University, Via Rita Levi Montalcini, 4, Pieve Emanuele, 20090 Milano, Italy; kamil.stankowski@humanitas.it (K.S.); stefano.figliozzi@humanitas.it (S.F.); vincenzo.battaglia@humanitas.it (V.B.); federica.catapano@hunimed.eu (F.C.); marco.francone@hunimed.eu (M.F.); 2 Humanitas Research Hospital IRCCS, Via Alessandro Manzoni, 56, Rozzano, 20089 Milano, Italy

**Keywords:** echocardiography, CMR, cardiovascular magnetic resonance, late gadolinium enhancement, speckle tracking, hypertrophy

## Abstract

Fabry disease (FD) is a genetic lysosomal storage disease with frequent cardiovascular involvement, whose presence is a major determinant of adverse clinical outcomes. As a potentially treatable cause of left ventricular hypertrophy (LVH) and heart failure with preserved ejection fraction, the early recognition of FD is crucial to initiate enzyme replacement therapy and improve long-term prognosis. Multimodality imaging plays a central role in the evaluation of patients with FD and helps in the differential diagnosis of other conditions presenting with LVH. In the present review, we explore the current applications of multimodality cardiac imaging, in particular echocardiography and cardiovascular magnetic resonance, in the diagnosis, prognostic assessment, and follow-up of patients with FD.

## 1. Introduction

Fabry disease (FD) is an inherited X-linked lysosomal storage disorder secondary to altered glycosphingolipid processing resulting from reduced or absent activity of the enzyme α-galactosidase A (aGAL), owing to a mutation in the *GLA* gene. The prevalence of FD in males is currently estimated at 1 in 3100 to 1 in 8500, significantly higher than previous estimates, which did not take into account recent newborn screening studies [1]. FD is a multi-system disease, and cardiovascular involvement is common and the leading cause of death and impaired quality of life.

The abnormal deposition of globotriasylceramide (Gb3) in the myocardium leads to left ventricular hypertrophy (LVH), diastolic dysfunction and eventually heart failure with preserved ejection fraction, conduction disturbances, atrial and ventricular arrhythmias leading to sudden cardiac death (SCD). Despite its relative rarity, the recognition of FD is necessary as one of the potentially reversible causes of LVH; however, FD remains underdiagnosed. The introduction of enzyme replacement therapy (ERT) has significantly changed the natural history of FD but also highlighted the need for early diagnosis to maximize its efficacy [2]. Multimodality cardiac imaging has a central role in each phase of the diagnosis and management of the condition and will be the scope of this review.

### 1.1. Pathophysiology

The deposition of Gb3 occurs in all cardiac cells and tissues, including myocytes, endothelial cells, fibroblasts, and conduction system cells, leading to systolic and diastolic dysfunction, conduction disturbances and arrhythmias. FD is not a simple storage cardiomyopathy, as experimental and clinical studies have shown that cardiac damage occurs secondary to Gb3 deposition, but inflammation and fibrosis also play a significant role, causing the biochemical and functional impairment of myocytes [3,4]. A seminal study by Nordin [5] proposed a three-stage model of cardiac FD progression. In the first stage, initially, subclinical myocardial accumulation starts in childhood and progresses faster in men than in women, leading to a lowering of native T1 values. In the second stage, LVH is more pronounced in men than in women, and inflammation ensues, causing the appearance of late gadolinium enhancement (LGE) and T2 elevation in the inferolateral basal segment accompanied by chronic troponin release. In the final stage, diffuse LGE with increasing T1 values, natriuretic peptide elevation, and overt heart failure dominate the clinical picture. Cardiovascular magnetic resonance (CMR) is able to capture each of the aforementioned alterations in the natural history of FD, allowing us to non-invasively characterize the myocardial alterations. In addition, according to Nordin [5], LVH observed in female patients is more likely secondary to a balance between sphingolipid accumulation and myocyte hypertrophy, whereas the LVH seen in male patients may be due to an increase in sarcomeric protein, leading to myocyte hypertrophy (triggered by sphingolipid accumulation).

### 1.2. Clinical Manifestations

Depending on mutation type and residual enzyme activity, two clinical forms exist: a classic early-onset type with renal, cardiac and nervous system involvement manifesting in childhood and a late-onset variant presenting with single organ involvement (usually cardiomyopathy or nephropathy). In women, according to the degree of skewed inactivation of the X chromosome, clinical manifestations are heterogeneous in terms of severity and organ involvement and usually appear a decade later than in men [6].

The diagnosis of FD in males is often based on the demonstration of the reduced or absent activity of the aGAL enzyme; in females, genotype confirmation is needed as the activity of the aGAL enzyme can be quite variable.

ECG changes classically associated with early-phase FD consist of bradycardia and a short PR interval (although present only in 14% of patients, according to a recent study [7]). The reduction in the difference between the PR interval and P wave duration (defined as PendQ) has been found to be more specific for accelerated atrio-ventricular conduction in early FD [8]. In the late-stage prolonged PR and QRS intervals, atrio-ventricular blocks, leftward axis deviation, high QRS voltage repolarization abnormalities (T-wave inversion) and QTc prolongation are common but not specific. An ECG-based nomogram, however, has demonstrated high accuracy in predicting FD involvement using CMR, as defined by low T1 values [9].

In the setting of unexplained left ventricular hypertrophy diagnosed using echocardiography, a standard ECG can help to differentiate FD from hypertrophic cardiomyopathy (HCM) [10]. Peculiar features of FD versus HCM ECG abnormalities are:

(1) In lead V1: a QRS complex with a right bundle branch block and an rSR’ morphology (FD) versus an rS complex with J point elevation in V1 (HCM);
(2) In lead aVF (augmented vector foot, in the frontal plane): ST-segment depression and a negative T wave (FD) versus a pathological Q wave and a discordant T wave (QT discordance) in HCM;
(3) A short PR interval (FD) versus a first-degree atrio-ventricular block (HCM);
(4) In lead aVL: a high R wave (>1.1 mV) and a large QRS (FD) versus a normal R wave and a narrow QRS (HCM).

Extra-cardiac manifestations of FD include, among other things, angiokeratomas, neuropathic pain, cornea verticillata, hypohidrosis, transient ischemic attack or stroke, renal failure, and hearing loss. Such extra-cardiac red flags should prompt further evaluation for FD in patients with suggestive imaging findings. In association with clinical and imaging assessment, biomarkers like troponin and natriuretic peptides are important for staging FD cardiomyopathy.

### 1.3. Management

In patients with Fabry disease, the goals of treatment are to avoid the development of LVH and fibrosis and, if this has already occurred, to prevent further progression and possibly reduce morbidity and mortality [11]. ERT with either agalsidase alfa or agalsidase beta has been developed for FD treatment; another therapeutic possibility is migalastat, a small-molecule oral chaperone active only in a proportion of *GLA* mutations. The treatment with ERT is costly and requires lifelong twice-a-month infusions; however, the early initiation of therapy is advocated as the progression of the disease may compromise its effectiveness. Criteria for initiation of ERT in FD patients have been defined: in particular, LVH (maximum wall thickness > 12 mm) without (or only minimal signs of) fibrosis and/or cardiac rhythm disturbances constitute a class I recommendation. On the contrary, treatment should not be initiated in FD patients with advanced cardiac disease with extensive fibrosis if only the heart is affected, as treatment is believed to be ineffective [12]. The management of patients with FD with cardiac involvement also includes the treatment of heart failure symptoms and brady- or tachyarrhythmias with a pacemaker or an implantable cardioverter defibrillator implantation [13].

## 2. Multimodality Imaging for Diagnosis

Cardiac imaging has proved fundamental in each aspect of Fabry cardiomyopathy. Echocardiography offers several techniques for the evaluation of patients with FD, ranging from standard two-dimensional transthoracic echocardiography and conventional Doppler to tissue Doppler and speckle-tracking methods. CMR has gained a leading role in FD as it is the reference modality for wall thickness and chamber size assessment (not requiring geometrical assumptions and good acoustic windows), regional and global function of the myocardium, or non-invasive tissue characterization in a wide spectrum of cardiomyopathies. CMR has also provided significant insights into the mechanisms that lead from Gb3 deposition to inflammation and fibrosis. Early recognition of FD in the context of unexplained LVH is crucial to initiate ERT and limit the progression of the disease. CMR can unveil subclinical myocardial involvement, help to non-invasively differentiate FD from other diseases manifesting with LVH, mainly HCM, and stage the progression of the disease. The role of cardiac imaging is of great importance, especially in patients with cardiac variant FD, as they may be lacking suggestive extra-cardiac involvement.

### 2.1. Echocardiography

**Figure 1:**
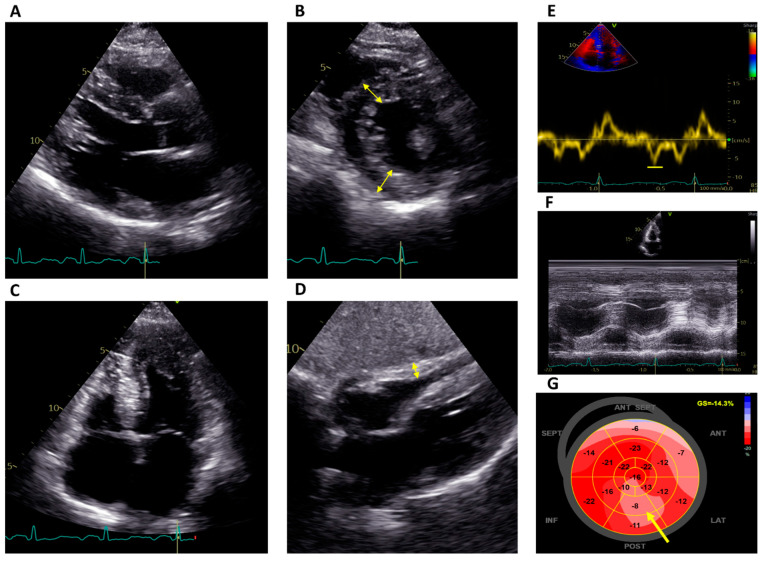
Echocardiographic features of a patient with Fabry disease on ERT. (**A**–**C**) Parasternal long-axis view, short-axis view at the level of the papillary muscles and four-chamber apical view, respectively, showing concentric left ventricular hypertrophy (double-edged arrows; septum 17 mm) with hypertrophy of the papillary muscles. (**D**) Subcostal four-chamber view demonstrating RV hypertrophy (double-edged arrow). (**E**) Reduced tissue Doppler septal strain e’ wave (6 cm/s). (**F**) Normal RV function (TAPSE 23 mm). (**G**) Reduced global longitudinal strain (−14.3%) with a regional reduction in the basal and mid inferolateral segments (arrow). ERT: Enzyme replacement therapy; RV: right ventricular.

Echocardiography is often the first imaging modality to be performed in suspected FD or to investigate otherwise unexplained ECG abnormalities, and it is also useful during the follow-up of affected patients. The hallmarks of FD are concentric LV remodeling or hypertrophy with the often-disproportionate hypertrophy of the papillary muscles; preserved systolic function, as measured by a left ventricular ejection fraction (LVEF) with an impaired myocardial strain; and right ventricular (RV) hypertrophy (Figure 1).

LVH is not specific to FD; it is present in other more common conditions, and other patterns of hypertrophy have been described, such as asymmetrical septal or apical hypertrophy, or even outflow tract obstruction (as in HCM). LVH is more common in male patients with FD, increases with age, and is associated with symptoms [14]. RV hypertrophy is present in 31–71% patients and the degree thereof correlates with LVH, without sex differences. RV systolic dysfunction, though, is rare [15,16].

Global longitudinal strain (GLS), a reliable marker of LV systolic function in a broad spectrum of cardiomyopathies, is an early marker of FD cardiomyopathy, potentially useful for identifying gene carriers before LVH becomes manifest [17,18]. In a study, a reduction in longitudinal strain was present in the basal segments of all FD patients with LVH and 50% of those not yet showing LVH, while it was absent in all patients of the control group [19]. In another study, despite no difference in LVEF between FD patients and healthy subjects, GLS was impaired in the first group (−16.5 ± 3.8% vs. −20.2 ± 1.7%), especially in those with LVH. Additionally, the longitudinal strain was most reduced in the basal segments of the LV in patients with FD [20]. Interestingly, a lower longitudinal strain in the basal inferolateral segment showed a correlation with the extent of LGE when using CMR [21]. A regional longitudinal strain worse than 12.5% was a strong predictor of fibrosis in that segment, while a regional longitudinal strain better than 16.5% could reliably exclude fibrosis [21]. The loss of a base-to-apex circumferential strain gradient, defined as the peak gradient difference between the averaged basal and apical strains, has been proposed as a specific finding in FD cardiomyopathy, regardless of the presence of LVH [22].

Diastolic dysfunction is common among patients with LVH and is sometimes observed even before LVH develops. Reduced tissue Doppler velocities, higher E/e’ ratios, and shortened isovolumetric relaxation times are early markers of cardiac involvement [23,24,25]. A restrictive filling pattern is rarely present and is usually associated with advanced cardiomyopathy. Similarly, left atrial (LA) enlargement and reduced atrial compliance have been described early in the course of the disease, in the pre-hypertrophic phase [26].

The “binary sign”, defined as a hyperechogenic stripe in the LV myocardium adjacent to a relatively hypoechogenic region, once considered an almost pathognomonic sign of FD [27], has been reconsidered in recent years [28,29]. Other reported findings in FD are aortic root dilatation and the thickening of the mitral and aortic valves secondary to the deposition of Gb3, although moderate or greater regurgitation is rare [30].

### 2.2. Cardiovascular Magnetic Resonance

**Figure 2:**
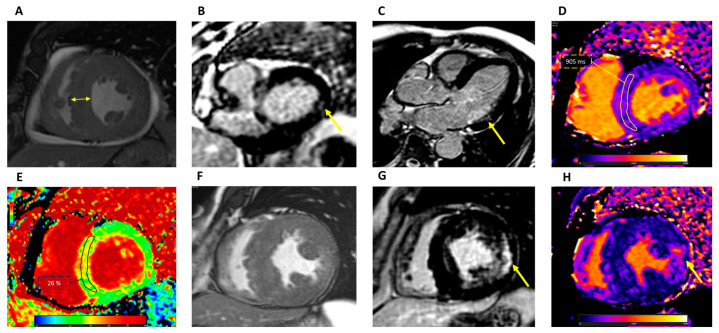
CMR findings in Fabry disease. (**A**) Cine short-axis view demonstrating concentric LV hypertrophy (double-edged arrow). LGE short-axis at the basal level (**B**) and three-chamber view (**C**) demonstrating intramural LGE in the basal inferolateral wall (arrow). (**D**) T1 mapping showing reduced intramyocardial T1 values: ROI 905 ± 35 ms at 1.5 T. (**E**) ECV map showing normal intramyocardial values: ROI 26 ± 3%. (**F**) Cine short-axis view demonstrating concentric LV hypertrophy and papillary muscles hypertrophy in a patient with advanced disease. (**G**) LGE short-axis view at the level of the papillary muscles showing extensive intramural LGE at the lateral segments and other foci in the septum and RV. (**H**) T1 mapping showing pseudo-normalization of T1 values (1021 ± 98 ms at 1.5 T) with areas demonstrating reduced values and areas, such as the one indicated by the arrow, corresponding to LGE, with focal elevation up to 1100 ms. CMR: cardiovascular magnetic resonance; LV: left ventricular; LGE: late gadolinium enhancement; ROI: region of interest; ECV: extracellular volume; RV: right ventricular.

CMR allows us to precisely identify and quantify left and right ventricular hypertrophy, including papillary muscle hypertrophy, and assess its spatial distribution (Figure 2).

CMR is more sensitive than echocardiography in detecting changes in LV mass [31,32]. LV mass is usually increased in FD patients; as discussed before, the contribution of LV papillary muscle mass to the total LV mass is disproportionately increased: from 8% in normal subjects to 20% in FD patients with LVH [33]. In a study, in patients with FD, papillary muscle hypertrophy was found even in the absence of LVH, supporting the notion that it is a helpful marker of FD [34]. The inclusion or exclusion of papillary muscles in the quantification of LV mass should always be specified in the CMR report, and appropriate reference values should be used.

LGE, representing areas of replacement fibrosis, is present in about 50% of patients [35]. The presence of LGE can aid in the diagnosis of FD, especially in the setting of LVH. LGE is initially focally distributed, and the most typical pattern of LGE distribution in the inferolateral basal-to-mid LV wall is mid-myocardial. As fibrosis develops, the thinning, hypokinesia, or akinesia of the same segments can sometimes be observed in advanced stages. However, other conditions, such as myocarditis, may also present with a similar LGE pattern. In patients with LVH with an atypical distribution for FD, LGE has been observed in the basal segment of the anterior septum and in the apical segments, resembling HCM. In patients with advanced disease, LGE tends to become extensive with a less specific appearance [36], and LVEF can be reduced.

A study by Niemann [37] found that the association between LGE and LVH varies depending on sex: in males, LVH precedes the development of fibrosis detectable through LGE imaging; on the contrary, in 23% of female patients, LGE was detectable even though LV wall thickness was normal, suggesting that the progression of the disease takes different pathways in males and females. LGE, thus, especially in female patients, is crucial for the detection of cardiac involvement and decisions on therapy initiation.

T1 mapping, a quantitative technique used to derive pixel-by-pixel T1 relaxation times of the myocardium, is particularly useful in FD. Likely due to the intramyocardial deposition of sphingolipids, native T1 values are lower than expected in other cardiomyopathies presenting with LVH, where native T1 is normal or, more frequently, increased, except for iron overload cardiomyopathy. Low T1 values can also testify to LV involvement before hypertrophy and fibrosis appear, allowing for an early diagnosis of cardiac involvement [38,39]. Low T1 values are present in 48% to 59% of patients without LVH, and an association has also been found with impaired LV-GLS [40]. A recent meta-analysis of 14 studies established that the weighted mean native T1 values were 984 ± 47 ms in FD patients and 1016 ± 26 ms in healthy subjects, with a pooled standardized mean difference of −2.38, and the degree of T1 shortening was influenced by the male sex and the presence of LVH [41]. As discussed before, sphingolipid deposition triggers inflammatory and profibrotic cellular pathways, which in turn tend to increase regional T1 values over time in areas of fibrosis (pseudo-normalization of T1 values); in the advanced stage of the disease, T1 values may be globally elevated due to diffuse fibrosis. Nonetheless, low T1 values in the context of LVH should prompt further evaluation for FD. On the other hand, normal native T1 values do not exclude FD cardiomyopathy, sometimes being noticed in untreated patients with mild LVH (especially females) or in advanced disease due to concomitant myocardial fibrosis. Interestingly, FD patients who have not yet developed LVH and detectable myocardial storage through T1 shortening still show lower T1 values, albeit in the normal range, compared to healthy controls, as well as subtly reduced LV-GLS, microvascular dysfunction and ECG abnormalities [42]. As such, the screening of FD gene carriers with GLS and T1 mapping seems a promising strategy to unmask the earliest signs of cardiac involvement.

Extracellular volume (ECV) is usually normal in FD except for areas with LGE, confirming that, at least in part, LVH is due to cell hypertrophy rather than extracellular matrix expansion [43].

T2 mapping shows elevated T2 values, both globally and in areas with LGE; this finding has been described [44] as a peculiar feature of FD ventricular hypertrophy, which can help in further differentiating sarcomeric HCM from FD phenocopies, thus supporting a pivotal role for inflammation in the pathogenesis of FD hypertrophy and disease progression toward myocardial replacement fibrosis.

Atrial function, expressed as an LA conduit strain and evaluated using feature tracking CMR, has been found to be already reduced in patients in the pre-hypertrophic phase, where only T1 mapping abnormalities were present [45]. This probably testifies to underlying atrial myopathy due to Gb3 deposition occurring earlier than LV remodeling.

The characteristic features of FD using echocardiography and CMR are summarized in Table 1.

### 2.3. Differential Diagnosis of Left Ventricular Hypertrophy

A hypertrophic phenotype may develop as an adaptive response to different stimuli, and CMR is key in the differential diagnosis of other diseases presenting with LVH, such as amyloidosis, HCM, aortic stenosis and hypertensive cardiomyopathy, or even LV hypertrophy in athletes, although a degree of overlap exists between these different conditions. FD is regarded as a phenocopy of HCM and, indeed, timely diagnosis is often missed as some patients with FD are misdiagnosed as having HCM and excluded from disease-modifying treatments. Accordingly, screening studies conducted in patients with HCM have found a prevalence of *GLA* gene mutations, especially those causing late-onset cardiac variants, of around 1% [46,47]. Similarly, in a series of patients undergoing surgical myectomy for LV outflow tract obstruction, a condition traditionally associated with HCM, a genetic analysis of the sample confirmed a *GLA* mutation in 1.3% of the patients [48]. These findings advocate for the systematic screening of FD in adult patients with the HCM phenotype, although it must be recognized that a subset of FD patients with late-onset variants have LVH that does not reach the 15 mm threshold for HCM diagnosis.

HCM sometimes can manifest with concentric hypertrophy, but the degree of hypertrophy is usually greater than with FD; in HCM, LGE and impaired regional strain are typically present in the most hypertrophied segments. In a study of 40 patients with LVH divided into 2 groups (FD and HCM) and matched for the degree of LVH and age, the FD group had lower LVEF, more reduced regional longitudinal strain in the inferolateral wall of the LV, and more impaired RV-free wall longitudinal strain, and the pattern of hypertrophy was more often concentric [49]. The finding of more profound subclinical RV impairment was confirmed in a recent study of 140 patients with FD or HCM and may be helpful in the differential diagnosis [50].

CMR-based studies showed that atrial remodeling, expressed as more pronounced LA dilatation and worse LA strain, is greater in HCM than in FD cardiomyopathy, with a good potential for distinguishing the two conditions [51]. Another study using 3T CMR found that a septal native T1 lower than 1220 ms distinguished patients with FD from HCM with an accuracy of 95%, providing incremental diagnostic value beyond age, sex, and conventional imaging features [52]. The association of myocardial hypertrophy with low native T1 values and elevated T2 values should be considered suggestive of FD-associated cardiac involvement (Table 2).

In cardiac amyloidosis, instead, native T1 values and ECV are globally increased, and LGE is often present with a global subendocardial or transmural pattern. LGE is less helpful in aortic stenosis and hypertensive cardiomyopathy, but elevated T1 values coupled with a globally reduced longitudinal strain in AS and regionally at the level of the septum in hypertensive cardiomyopathy are valuable clues.

## 3. Cardiac Imaging for Prognostic Stratification and Monitoring Treatment Response

### 3.1. Prognostic Stratification

Despite being a multi-system disease, FD can be regarded as a predominantly cardiac disease from a mortality perspective since most deaths are SCDs [59]. As discussed before, echocardiography and CMR are commonly used to unmask cardiac involvement in FD, but their role in predicting adverse outcomes is somewhat less clear. The identification of imaging markers able to predict adverse cardiac events, however, is of great interest to identify high-risk patients and possibly offer intensified follow-up and earlier access to the available therapies.

#### 3.1.1. Echocardiography

The prognostic role of LV-GLS has been confirmed in a study by Spinelli et al. [60], where a less negative LV-GLS conferred a higher risk of adverse outcomes. Also, a reduced basal longitudinal strain was associated with major adverse cardiovascular events at a 7-year follow-up [20].

As discussed above, RV hypertrophy is common in FD patients. Graziani et al. [16], in a study of 45 genetically confirmed FD patients followed up for an average of 51 months, found that 31.1% of patients had RV hypertrophy, while RV systolic function, defined as TAPSE and S’ TDI, was normal in all cases. In univariate analysis, both RV hypertrophy and impaired systolic function were associated with adverse outcomes; however, in multivariate analysis, the association was lost. A study by Meucci et al. [61], investigating the prognostic power of RV-FWLS, failed to show an independent association with adverse outcomes after adjusting for LV-GLS and LAVI, suggesting that prognosis is mainly influenced by left-sided parameters. However, caution is needed as these studies have been limited by small sample sizes and short follow-up times. Further research is needed to exclude any prognostic significance of RV involvement in FD.

The extent of hypertrophy, indeed, seems to be associated with outcomes. In a prospective study of 78 patients, a maximum LV wall thickness > 20 mm measured using echocardiography was found in all subjects with ventricular arrhythmias [62]. Elevated LV filling pressures, as assessed by the E/e’ ratio, have also been associated with unfavorable prognosis [63]. Other parameters, assessed by echocardiography, have not been studied as potential prognostic factors in FD.

#### 3.1.2. Cardiovascular Magnetic Resonance

CMR, LVH and LGE have been found to be potent prognostic factors in FD. In a study [64] involving 90 patients, with a median follow-up of 3.6 years, patients with LVH had a three-times higher risk of adverse cardiac events (HR 3.0; 95% CI: 1.1–8.1), defined as ventricular tachycardia, bradycardia requiring device implantation, severe heart failure, and cardiac death. Additionally, a continuum has been found between increased LV mass and outcome, as a 5 g/m^2^ increase in indexed LV mass was linked to an 8% increase in the risk of adverse outcomes. Although the contribution of papillary muscles and trabeculations to LV mass is disproportionately elevated in FD patients compared to controls [33], it was demonstrated that excluding papillary muscles and trabeculations results in a slightly better predictive value of adverse prognosis as well as better reproducibility [65].

In a study mentioned previously [64], patients with LGE had a seven-fold increased risk of adverse outcomes compared with those without LGE (HR 7.2; 95% CI: 1.5–34). The risk also increased with LGE extent, with a 5% increase in LGE extent associated with a 44% increase in risk. Patients with an extent of LGE 15% or greater of LV mass—the same threshold currently used in HCM patients—were at the highest risk of adverse events (12-times higher than those without LGE). Of note, the impact of LVH and LGE on prognosis was not influenced by sex. In another prospective study of 73 patients followed up for a median of 4.8 years, 13 patients had ventricular arrhythmias documented using ambulatory Holter monitoring, all of whom showed LGE, whereas none of the patients without LGE had evidence of ventricular arrhythmias. Five SCDs occurred in patients with LGE and prior ventricular arrhythmias. Moreover, the increase in LGE over serial imaging was greater in those patients with ventricular arrhythmias, and using multivariate regression, it was found to be the only predictor of ventricular arrhythmias identified [66].

From tissue characterization, lower native T1 values were found to be associated with clinical worsening at 12 months’ follow-up in a group of patients with genetically confirmed FD and no evidence of LVH [67]. Elevated regional T2 values, i.e., in the basal inferolateral wall, were found to be associated with troponin release (a marker of chronic myocardial injury), global longitudinal strain impairment and clinical worsening at 12 months’ follow-up in a multi-center prospective study involving 186 consecutive FD patients [44]. As discussed before, unlike T1 values, which may show a biphasic course in FD patients, T2 values increase linearly during the progression of the disease, potentially representing a more clinically useful marker of disease activity.

### 3.2. Monitoring Treatment Response

Multimodality imaging can potentially assist the treating physician after the initiation of ERT. Although the evidence is derived from mostly retrospective observations, it seems that imaging features assessed by echocardiography or CMR are able to predict the extent of response to available treatments.

#### 3.2.1. Echocardiography

Echocardiography is frequently used for the longitudinal monitoring of patients with FD after treatment initiation, ideally every 6 to 12 months. Numerous studies have been conducted on markers of LVH, such as LV mass and LV wall thickness, showing conflicting results, with some studies showing progression despite therapy [68] and others showing the stabilization of [69] or reduction in LVH after treatment, especially in specific cohorts of patients, such as those with baseline LVH [70,71]. A meta-analysis found that LV mass increased in male patients, although at a lower rate than in untreated patients, while it decreased in female patients with baseline LVH and remained stable in female patients without baseline LVH [72]. Probably, the timing of therapy initiation is a key factor in predicting treatment response.

Other studies have demonstrated improvement in diastolic function: a decrease in the E/e’ ratio and LV filling pressures [68] and an increase in the LA strain using speckle-tracking imaging, with a concomitant reduction in LA volume [73], but no improvement in LV global longitudinal strain has been demonstrated so far [74].

#### 3.2.2. Cardiovascular Magnetic Resonance

Randomized data show that LV mass was significantly reduced after 6 months of treatment with agalsidase alpha: the mean decrease in indexed LV mass was 6.4 g/m^2^ in the treatment arm, while it was 12 g/m^2^ in the control arm [75]. Another prospective study [76] demonstrated significant reductions in LV mass and LV wall thickness as well as native T2 after about 2 years of treatment with agalsidase beta.

LGE is probably the most potent predictor of response to ERT. Beer at al. [77] demonstrated that only patients without LGE had significant reductions in LV mass at follow-up. The amount of LGE increased despite treatment; however, no patients without LGE at baseline developed LGE after the initiation of ERT. Another study [66] did not find reductions in LGE in treated patients, suggesting that LGE, a sign of more advanced stages of the disease, represents myocardial fibrosis unlikely to be influenced by ERT, thus calling for dedicated treatments.

An increase in native T1 relaxation time has also been observed in treated patients, potentially signaling a reduced accumulation of sphingolipids in the myocardium [74], while no impact was found on RV hypertrophy and function after ERT in a prospective study of FD patients followed up for a median of 3 years [15].

## 4. Conclusions

FD is one of the genetic diseases for which treatment is available and potentially capable of improving prognosis. Multimodality imaging allows for a comprehensive evaluation of cardiac involvement and is fundamental when the disease is in the subclinical phase, for diagnosis, prognostic assessment, and the evaluation of response to available therapies. CMR is the imaging technique of choice due to its ability to characterize myocardial tissue and provide reproducible measurements of LV mass. Echocardiography with strain imaging efficiently complements CMR. Further, longitudinal studies are needed to prospectively validate the proposed prognostic markers of FD cardiomyopathy, to find reliable imaging findings to trigger the initiation of therapy, and to monitor its progression over time.

## Figures and Tables

**Table 1:** Fabry disease features assessable using echocardiography, CMR or both.

Echocardiography	CMR
Concentric LV hypertrophy with disproportionate hypertrophy of the papillary muscles [33]	
Elevated LV mass [14]	
Right ventricular hypertrophy with normal systolic function [15,16]	
Reduced tissue Doppler strain [23]	Basal inferolateral LGE [35]
Reduced GLS [17,18]	Reduced global native T1 (may be normal or increased in advanced stages) [38,39]
Abnormal basal inferolateral regional strain [19]	Normal ECV (may be increased in advanced stages) [43]
Loss of basal-to-apex circumferential strain gradient [22]	Elevated T2 in the basal inferolateral wall [44]
LA dilation (not specific to FD) [26]	

CMR: cardiovascular magnetic resonance; LV: left ventricular; LGE: late gadolinium enhancement; GLS: global longitudinal strain; ECV; extracellular volume; LA: left atrium.

**Table 2:** Differential imaging features between Fabry disease and hypertrophic cardiomyopathy.

	Fabry Disease	Hypertrophic Cardiomyopathy
Left ventricular hypertrophy	Usually concentric [49]	Usually asymmetrical, apical or segmental [53]
Papillary muscles	Disproportionately hypertrophied [33]	Sometimes apically displaced [54]
Late gadolinium enhancement	Mid-wall or subepicardial basal inferolateral [35]	Mid-wall in most hypertrophied segments and RV insertion points [53,55]
T1 mapping	Global native T1 reduced; normal or elevated in advanced disease [38,39]	Normal or elevated T1 values [52,56]
Extracellular volume	Generally normal [43]	Generally normal or elevated [57]
T2 mapping	Elevated values in the basal inferolateral wall [44]	Sometimes elevated values in severely hypertrophied segments [57]
Global longitudinal strain	Usually impaired in the basal inferolateral wall [19]	Impaired in areas of hypertrophy [58]

RV: right ventricular.

## Data Availability

Data sharing not applicable.

## Abbreviations

FD	Fabry disease
aGAL	α-galactosidase A
Gb3	Globotriaosylceramide
LVH	Left ventricular hypertrophy
SCD	Sudden cardiac death
ERT	Enzyme replacement therapy
LGE	Late gadolinium enhancement
CMR	Cardiovascular magnetic resonance
HCM	Hypertrophic cardiomyopathy
LV	Left ventricular
LVEF	Left ventricular ejection fraction
RV	Right ventricular
GLS	Global longitudinal strain
ECV	Extracellular volume
LA	Left atrium
